# DETECTX: a novel deep learning approach for detecting pneumonia, tuberculosis, and aspergillosis from chest X-rays

**DOI:** 10.3389/fcimb.2026.1915083

**Published:** 2026-09-17

**Authors:** Xian-Hong Wang, Naeem Ahmed, Muhammad Saeed, Hassan Ali, Guang-Wei Chen, Shi-Chang Sun, Wei-Xia Dong, Tie-Shan Teng, Zhong-Xia Liao, Umair Ali khan Saddozai

**Affiliations:** 1 Department of Internal Medicine and Pediatrics, Medical School, Henan Provincial Research Center of Engineering Technology for Nuclear Protein Medical Detection, Zhengzhou Health College, Zhengzhou, Henan, China; 2 School of Software, Nanjing University of Information Science and Technology, Nanjing, China; 3 College of Computer Science and Technology, Nanjing University of Aeronautics and Astronautics, Nanjing, China; 4 Department of Pulmonary and Critical Care Medicine, People’s Hospital of Fangcheng County, Nanyang, Henan, China; 5 Department of Microbiology and Immunology, Medical School; Henan Provincial Research Center of Engineering and Technology for Medical Detection of Nuclear Protein, Zhengzhou Health College, Zhengzhou, Henan, China; 6 Kaifeng Key Laboratory for Infection and Biosafety, School of Basic Medical Sciences, Henan University, Kaifeng, Henan, China; 7 Department of Health Management, ZhengZhou Health College, Zhengzhou, Henan, China; 8 Institute of Translational Medicine, Medical College, Yangzhou University, Yangzhou, Jiangsu, China

**Keywords:** aspergillosis, deep learning, disease detection, medical image classification, pneumonia detection using x-ray images, siamese CNN, tuberculosis

## Abstract

**Background:**

Aspergillosis, pneumonia, and tuberculosis are distinct respiratory illnesses, but they share similar symptoms, including cough, fever, and shortness of breath. This overlap can make accurate diagnosis challenging. Delayed or incorrect identification, particularly of aspergillosis, can lead to worsening patient outcomes and significant lung damage.

**Methods:**

To address this diagnostic challenge, we developed a deep learning based system designed to differentiate between aspergillosis, pneumonia, tuberculosis, and healthy lungs using chest X-ray images. We implemented three models: Convolutional Neural Networks (CNN), ResNet-50, and a novel Siamese Convolutional Neural Network. The models were trained on a dataset of 7,200 images, categorized into four groups: aspergillosis, pneumonia, tuberculosis, and healthy. We utilized various training-testing ratios to optimize performance, which was evaluated using accuracy, precision, recall, F1-score, and confusion matrices.

**Results:**

Among the models tested, the Siamese neural network outperformed the others, achieving an accuracy of 98.72%, surpassing the performance of state-of-the-art models in comparative studies.

**Conclusion:**

The novel Siamese CNN model demonstrated excellent accuracy in differentiating respiratory diseases from chest X-ray images, offering a promising tool for more accurate and timely diagnoses. We also developed an intuitive web interface that enables healthcare providers to upload and analyze medical images, facilitating quicker and more reliable diagnoses to support timely interventions.

## 1 Introduction

Pulmonary diseases are a significant contributor to global mortality and morbidity, placing considerable strain on medical healthcare services (Santosh et al., 2022c). Chest radiography (X-ray), valued for its effectiveness in detecting and characterizing cardiothoracic and pulmonary issues, remains the most widely utilized imaging technique (Yu-Xing et al., 2020). Pneumonia, defined by inflammation of the air sacs in one or both lungs, leads to symptoms such as cough, fever, chills, and breathing difficulties due to pus accumulation (Singh and Tripathi, 2022). Early and accurate screening for pneumonia is a critical step in its diagnosis, enhancing radiologists’ productivity and preventing delays in treatment (Ahmed et al., 2023). Similarly, screening for lung abnormalities is essential in the diagnostic process for lung infections, improving clinical efficiency and ensuring timely interventions (Wang et al., 2020; Henderson and Santosh, 2022). Common diagnostic tools for pneumonia include chest X-rays, CT scans, and MRI imaging, with chest X-rays being the most accessible (Ling and Cao, 2019). However, the accuracy of diagnosing pneumonia from chest X-rays depends heavily on the medical expert’s expertise, and the close similarity of imaging patterns across pulmonary diseases can lead to misdiagnosis, even by skilled physicians, potentially causing severe and long-lasting consequences.

Efforts to address the challenge of detecting lung abnormalities have extensively utilized machine learning and deep learning frameworks, including CNNs, support vector machines (SVM), random forests (RF), logistic regression (LR) and k-nearest neighbors (KNN). CNN-based feature extraction has been particularly prominent in the literature for its effectiveness in medical image analysis. In (Yu-Xing et al., 2020), researchers evaluated the performance of deep CNN architectures, such as AlexNet, GoogLeNet, VGG, DenseNet, and ResNet, in distinguishing normal from abnormal chest X-rays, using radiologist labels and expert consensus for validation. Their study trained and tested these models on a diverse dataset to assess classification accuracy. Similarly (Mahbub et al., 2022), proposed a tailored deep neural network to identify tuberculosis (TB), pneumonia, and Aspergillosis from chest X-rays, employing cross-validation across datasets to evaluate performance for non-healthy cases. A hybrid deep learning approach, VGG Data STN with CNN (VDSNet), was introduced in Tuberculosis detection has also seen notable advancements (Qin et al., 2023). Qin et al. (2023) presented a deep-learning ensemble for tuberculosis and pneumonia detection from chest X-rays. Chen et al. (2024) developed a deep neural network using Google Teachable Machine, achieving a 92% area under the curve (AUC) on multi-country CXR datasets. However, their model exhibited variability in distinguishing TB from pneumonia-like patterns. Gurung et al. (2023) proposed a two-stage pipeline combining UNet segmentation and Xception classification with Grad-CAM visualization, yielding 95% accuracy on the Montgomery and Shenzhen datasets. In (Liang and Zheng, 2020), a deep learning system for diagnosing pediatric pneumonia was developed, demonstrating success with an image dataset. (Jaiswal et al., 2019) described a deep learning method for pneumonia detection using chest X-rays, using Mask RCNN for classification and detection, achieving notable adaptability and effectiveness.

Shakeri and Far (2024) created a transfer learning approach to the pneumonia detection task with DenseNet121, DenseNet169 and ResNet101 on 5,863 chest X-ray images. The proposed DenseNet121 archived the highest validation accuracy of 98.68% with a validation loss of 0.04, and test accuracy of 94%, 93% AUC, 93% precision, 98% recall and 95% F1-score. Although the study showed that transfer learning can be effective for pneumonia diagnosis, it only showed that pneumonia can be diagnosed, and it was a binary problem where pneumonia could only be distinguished from non-pneumonia.

Lakhani and Sundaram (2017) designed a deep learning system for automatic pulmonary TB classification from chest radiographs, where the convolutional neural networks were based on the AlexNet and GoogLeNet architectures. The models were trained and validated on several publicly available chest X-ray datasets, and showed strong diagnostic performance, with AUCs greater than 0.99 and high sensitivity and specificity for TB detection. The proposed framework was created for the binary classification of tuberculosis and was not used for simultaneous classification of all other respiratory diseases with similar radiographic appearances, only for comprehensive multi-class clinical diagnosis.

Hwang et al. (2016) presented a tuberculosis screening framework for automatic analysis of chest X-rays that was built using deep convolutional neural networks and transfer learning. The three real-world TB screening datasets yielded AUC scores of 0.96, 0.93 and 0.88 respectively, proving the ability of deep CNNs to serve large-scale TB screening tasks. The framework was designed only for binary tuberculosis detection without considering multi-class discrimination among respiratory diseases.

Maheswari et al. (2024) introduced an explainable shallow CNN for TB screening, achieving a 94% F1-score with reduced parameters, but it underperformed on subtle cavitary lesions. DETECTX improves upon these by integrating attention-driven feature extraction to enhance differentiation of TB from visually similar conditions, such as aspergillosis. Another research demonstrated reliable pneumonia identification. CheXNet, a 121-layer CNN, was used in (Rajpurkar et al., 2017) to detect 14 diseases from 110,000 images, validated with radiologist-annotated test data. In (Hwang et al., 2016), a deep learning model for chest X-ray abnormality detection was evaluated using cross-population train/test data from the Montgomery County and Shenzhen datasets, achieving accuracies of 76.05% and 71.47% with ResNet-101 (Maheswari et al., 2024). To replicate clinical pneumonia screening (Rajpurkar et al., 2017), proposed a deep regression model validated on 900 clinical cases, integrating multi-modal data and multi-channel images. In (Das et al., 2020), a deep neural network detected circular foreign objects in chest X-rays, achieving 97% accuracy, 90% recall, and 93% F1-score.

For binary classification (pneumonia vs. normal) (Santosh, 2020a), utilized X-ray and CT scans with transfer learning CNNs (SqueezeNet, VGG16, VGG19, AlexNet, and Inception V3) to extract features, followed by classification with KNN, SVM, LR, naive Bayes (NB), and artificial neural networks (ANN). Their results showed Inception V3 achieving 97% accuracy. In (Qian et al., 2020), an end-toend CNN model for tuberculosis screening outperformed other deep learning methods, reaching 91.7% accuracy. (Santosh et al., 2020) developed a computer aided diagnostic system combining DenseNet-121, GoogleNet, and ResNet18, achieving 98.81% accuracy on Kermany and RSNA datasets using fivefold cross-validation (Muhammad et al., 2021) and comparing 15 CNN architectures for pneumonia detection, selecting the model with minimal loss and highest accuracy. Aspergillosis detection from CXRs remains underexplored due to its rarity and diagnostic complexity (Fu et al., 2024). Siddiqi and Javaid (Das et al., 2021) noted that aspergillosis is often subsumed under general fungal pneumonia categories, with few targeted deep learning models. Rahman et al. (Kundu et al., 2021) developed a multi-disease detection framework for pneumonia, COVID-19, and TB using ensemble CNNs, achieving 98.7% accuracy but excluding aspergillosis due to limited data availability. Tootooni et al. (Ali et al., 2026) proposed a knowledge distillation model for triaging COVID-19, pneumonia, and TB, reporting 97% sensitivity, yet fungal diseases like aspergillosis were not addressed. A review by Rahman et al. (Siddiqi and Javaid, 2024) emphasized the need for inclusive multi-disease models, highlighting that aspergillosis-specific features, such as halo signs, are rarely extracted in existing frameworks. To overcome these diagnostic challenges, artificial intelligence (AI)-based strategies offer a robust solution by delivering more accurate and reliable results (Rahman et al., 2023b; Tootooni et al., 2024).

The field of chest X-ray analysis for pulmonary aspergillosis is still relatively unexplored in comparison to pneumonia and tuberculosis, due to the scarcity of annotated datasets and the significant similarity between the radiography of pulmonary aspergillosis and other pulmonary infections (Bhapkar et al., 2020; Santosh and Ghosh, 2021; Rahman et al., 2023a). Unlike the current studies, the proposed DETECTX framework explicitly includes aspergillosis in a four-class classification model along with four other classes of chest radiographs: pneumonia, tuberculosis, aspergillosis, and normal. Furthermore, the Siamese convolutional network incorporates contrastive feature learning and squeeze and excitation attention to boost discriminative feature representation, and the built-in clinical decision-support interface further strengthens its applicability in real-world clinical care. The proposed framework was extensively tested with conventional machine learning algorithms and state-of-the-art deep learning algorithms on standard performance metrics: accuracy, precision, recall, F1 score, and AUC. The experimental results showed that DETECTX can achieve an accuracy of 98.72%, significantly higher than the existing methods, and it can also have good generalization performance for visually similar pulmonary diseases (**Figure 1**). Moreover, an easy-to-use web-based clinical decision-support interface has been designed to enable automated diagnosis, treatment recommendation and consultant matching, enhancing the framework’s usability in real world health systems. The main contributions of this study are summarized as follows:

**Figure 1:**
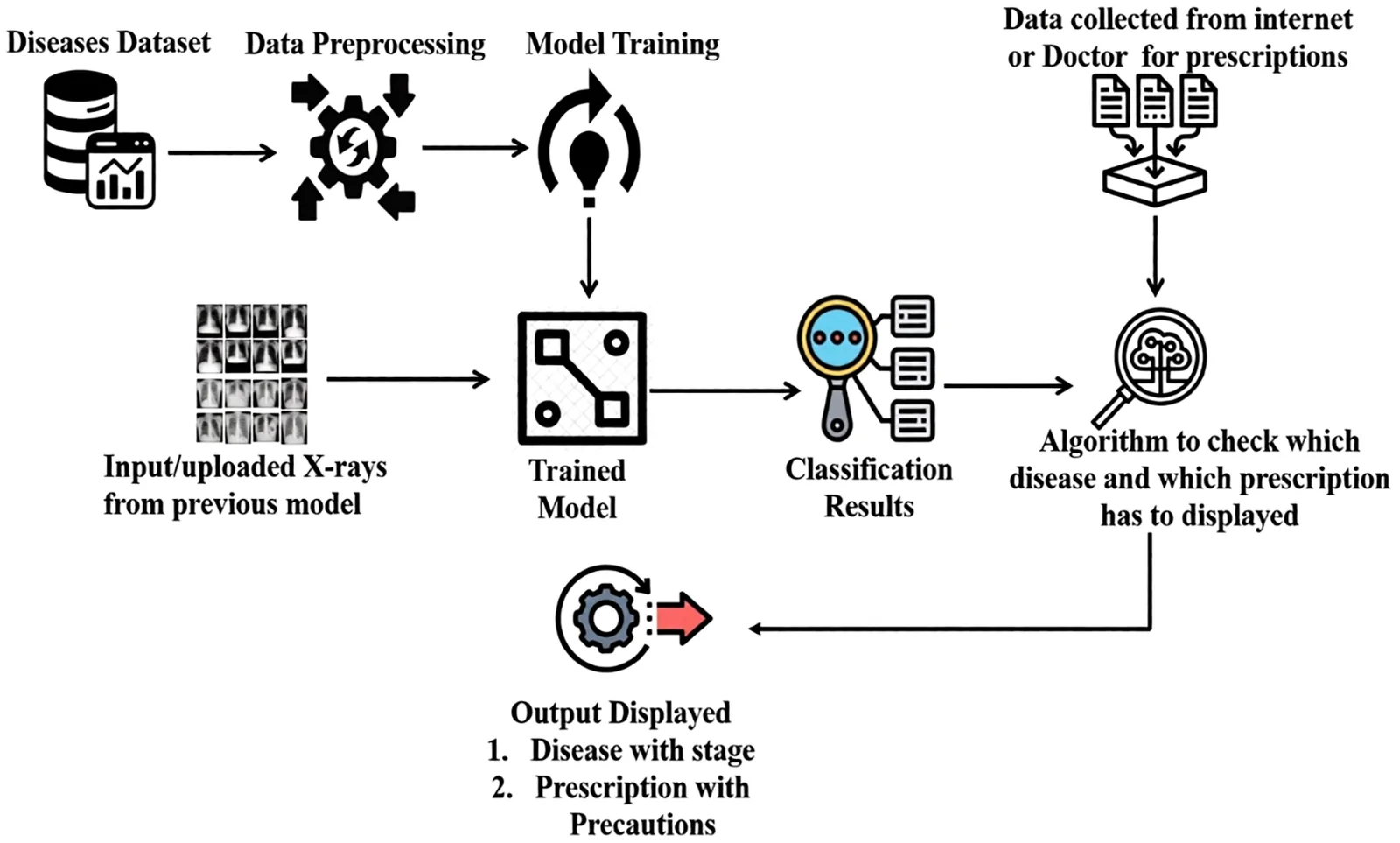
The Disease Classification Model: This model preprocesses chest X-ray images (normalization, resizing, augmentation) and uses deep learning architectures such as Siamese CNN, CNN, and ResNet-50 for disease diagnosis. A user-friendly web interface allows for efficient input and classification of respiratory diseases, followed by personalized recommendations for treatment and specialist matching.

- A unified deep learning framework, DETECTX, was developed for the automated four-class classification of pneumonia, tuberculosis, aspergillosis, and normal chest radiographs using a Siamese convolutional neural network.
- A Siamese convolutional neural network integrated with contrastive feature learning and squeeze-and-excitation attention was proposed to enhance discriminative feature representation and improve the classification of pulmonary diseases with similar radiographic characteristics.
- A comprehensive experimental evaluation was conducted by comparing DETECTX with conventional machine learning algorithms and state-of-the-art deep learning models using standard performance metrics, demonstrating superior diagnostic performance.
- A user-friendly web-based clinical decision-support interface incorporating automated diagnosis, treatment recommendation, and consultant matching was implemented to improve the practical applicability of the proposed framework in real-world healthcare environments.

The rest of this paper is structured as follows: Section 2, provides an overview of the dataset, preprocessing techniques, and the methodologies employed, including CNN, ResNet-50, and the proposed Siamese model. Section 3, presents the performance evaluation, and comparative analysis. Finally, Section 4 concludes the paper by summarizing the key findings and offering recommendations for future work.

## 2 Materials and methods

In this study, we developed a comprehensive Disease Classification Model, as illustrated in **Figure 2**, designed to accurately diagnose respiratory diseases such as pneumonia, tuberculosis, and Aspergillosis using chest X-ray images. The proposed system begins with preprocessing a dataset of chest X-ray images to ensure data quality and compatibility with our models. This preprocessing involves steps such as normalization, resizing, and augmentation to enhance model robustness (Mukherjee et al., 2020; Santosh, 2020b; Randieri et al., 2025). The preprocessed dataset is then used to train our novel Siamese CNN model, alongside other architectures like CNN and ResNet-50.

**Figure 2:**
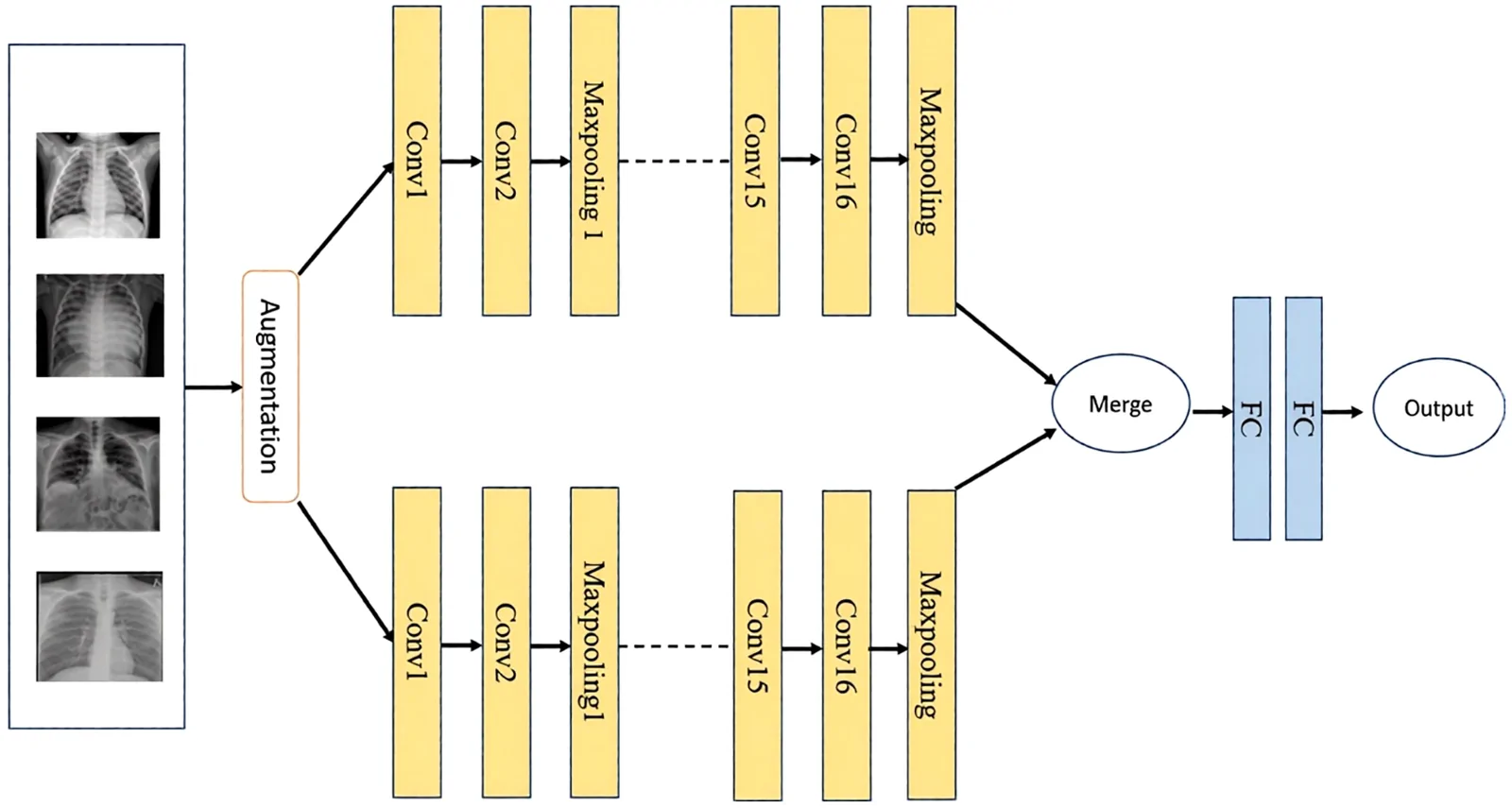
overview of the proposed model architecture. The model processes four types of chest X-ray images (normal, pneumonia, Aspergillus, and tuberculosis) through parallel processing pathways, with feature extraction from convolution layers and max-pooling layers.

Chest X-ray images provided by users are input into the trained Disease Classification Model through a user-friendly web interface. The model analyzes these images to classify the presence of a specific respiratory disease. Following classification, an integrated algorithm, informed by insights from medical professionals and curated online medical resources, determines the disease’s identity, its stage, and appropriate clinical recommendations. The system outputs a detailed report displaying the diagnosed disease name, its stage, a tailored prescription, and recommended precautions to guide patient care.

### 2.1 Data gathering

For this study, we sourced a comprehensive dataset from the open-access Kaggle repository to support the development and evaluation of our novel Siamese convolution neural network model for respiratory disease classification (Patel, 2020; Santosh and Ghosh, 2022; Santosh et al., 2022b; Santosh et al., 2022a). The dataset comprises 7,200 chest X-ray images, systematically categorized into four classes: Aspergillosis, normal, pneumonia, and tuberculosis. Specifically, the dataset includes 1,850 images of Aspergillosis cases, 1,600 images of normal (healthy) cases, 1,950 images of pneumonia cases, and 1,800 images of tuberculosis cases. A detailed breakdown of the dataset’s composition is presented in **Table 1**, summarizing the number of images per class. Additionally, representative samples of the chest X-ray images across these categories are illustrated in **Figure 3**, providing a visual reference for the dataset’s characteristics.

**Table 1:** Dataset distribution and stratified data partitioning of DETECTX dataset.

Class	Total images	Training (70%)	Validation (15%)	Independent test (15%)
Normal	1600	1120	240	240
Pneumonia	1950	1365	293	292
Tuberculosis	1800	1260	270	270
Aspergillosis	1850	1295	278	277
**Total**	**7200**	**5040**	**1081**	**1079**

Bold values indicate the total number of images and partitioned samples summed across all four disease classes.

**Figure 3:**
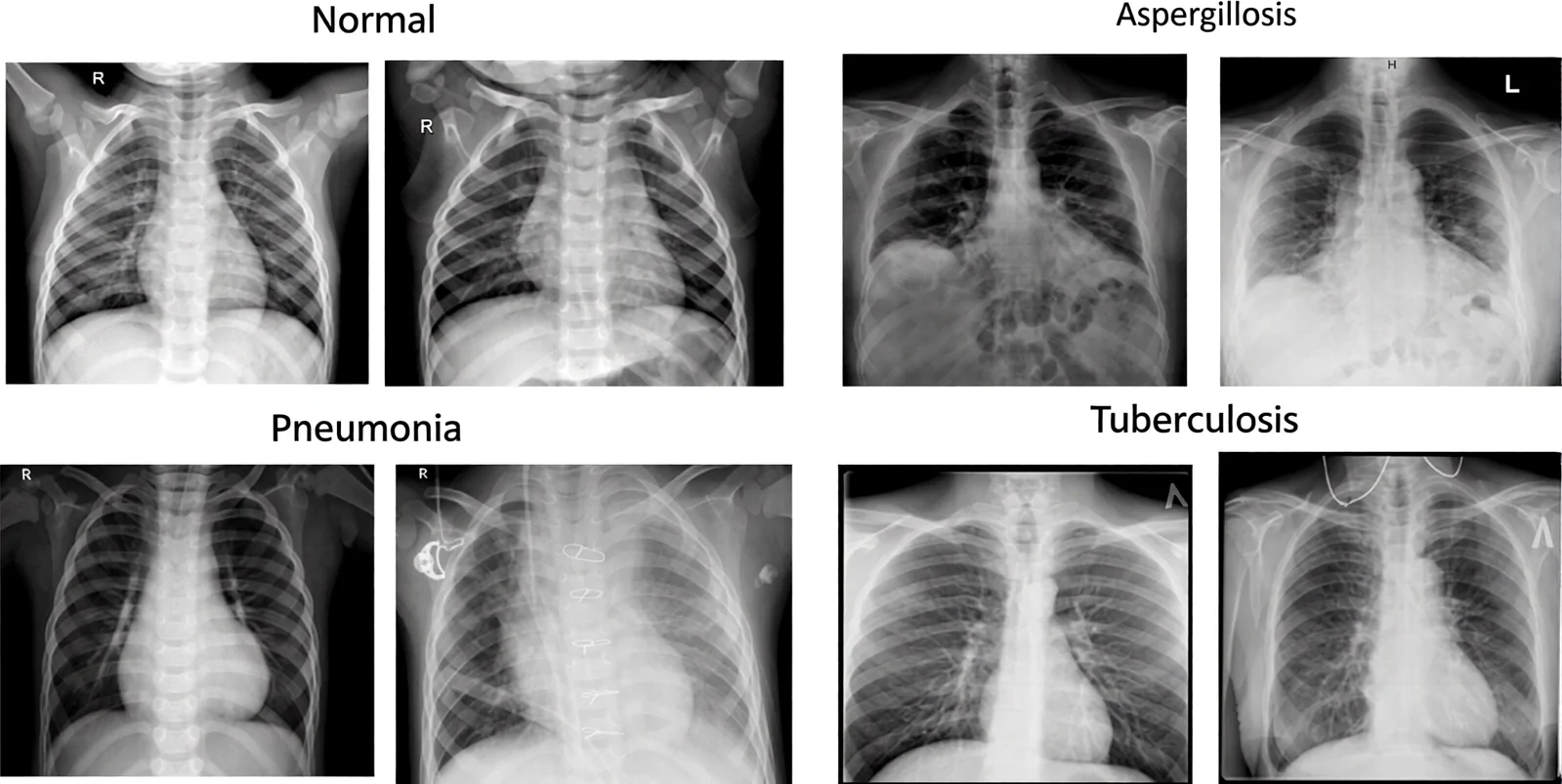
Representative chest X-ray images from the dataset: Samples of images from the categories of normal, aspergillosis, pneumonia, and tuberculosis. These images provide a visual representation of the dataset used for training and testing the disease classification model.

The data set used in this study was obtained from three public Kaggle repositories from which pictures were obtained from various clinical and institutional sources (JtiptJ, n.d.). The aspergillosis subset was composed anonymized radiological archives of patients with confirmed pulmonary aspergillosis (culture/histopathology) (Santosh et al., 2022b). The pneumonia subset was also collected by Kaggle repository (Santosh et al., 2022a) which consists of anteroposterior chest radiographs from children with bacterial or viral pneumonia. The TB datasets was collected from another Kaggle repository and have been well validated in the literature (Santosh and Ghosh, 2022) and consist of geographically and demographically diverse patients. The same criteria for the selection of images were given to all classes. Only pictures in high resolution JPEG (256x256 or higher) formats were retained. Marked motion artifacts and foreign body overlaps not due to study diseases. Every image had been given an unique id and a perceptual hash (pHash) was used to identify and remove near duplications. The deduplication procedure showed that there were no duplicate or near-duplicate image pairs for any of the training, validation, and test partitions, negating the issue of test set contamination due to repeat images. The image multiplicity per patient analysis was not made on the patient level because the images are only anonymized and not provided with patient information in the Kaggle repositories from which they were obtained. To reduce the risk that several images from the same patient were split into more than one partition, images were stratified when they were split by the sub-repository that contributed them and assigned to one of the partitions in a deterministic way.

This dataset serves as the foundation for training and testing our Disease Classification Model, enabling accurate detection and classification through the user-friendly web interface developed in this study. The distribution of the whole dataset and stratification into training, validation and independent test subsets are given in **Table 1**. Split was done without altering the original class distribution to avoid sampling bias.

### 2.2 Data preprocessing and augmentation

To prepare the dataset for training our novel Siamese convolution neural network model and other architectures, such as CNN and ResNet-50, all chest X-ray images were resized to a uniform dimension of 256 × 256 pixels. The 256 × 256 resolution was chosen because it represents the smallest common size across the datasets used in this study, accommodating the majority of images that were equal to or larger than this dimension. A complete augmentation pipeline was created to boost the diversity of the training set and enhance the robustness of the model to acquisition-related variability. All Augmentation operations were only applied to the training partition and not to the validation or test partitions. The mini-batches were produced online via the following transformations: Random rotation: within the range of [-15°; +15°] with bicubic interpolation and constant-value padding; Random horizontal flip with a probability of 0.5; Random vertical flip with a probability of 0.3; Width and height shift: in the range of 10% of the image dimension; Zoom augmentation: in the range [0.85; 1.15]; Gaussian noise injection: with a mean of 0 and a standard deviation sampled uniformly from [0; 0.02] to simulate sensor noise; Random brightness adjustment: in the range [0.8; 1.2] to simulate inter-scanner exposure variability. Together, these operations mimic the spectrum of image variation that can be expected from the differences in patient positioning, scanner calibration, and acquisition protocol, minimizing the chances of overfitting to the characteristics of the images.

### 2.3 Proposed siamese convolution neural network

The proposed SCNN-based framework follows a structured pipeline consisting of three main stages: (i) data preprocessing and augmentation, (ii) feature extraction using a Siamese convolutional architecture, and (iii) multiclass classification using fully connected layers. This step-wise design ensures efficient learning of discriminative features while maintaining robustness across different chest X-ray patterns. The novel SCNN developed in this study, drawing inspiration from the VGG16 architecture, is designed to enhance the diagnosis and classification of respiratory disorders. The SCNN architecture features two parallel modified VGG16 layers, termed “Siamese,” which are interconnected through a concatenation mechanism at each layer (**Table 2**). This design reduces the dependency on large datasets. By using 3-D views of the dataset, our approach achieved superior performance compared to state-of-the-art methods, particularly on small-scale chest X-ray images. The overview of the proposed model is illustrated in **Figure 2**.

**Table 2:** Detailed specifications of the SCNN architecture, including layer names, kernel sizes, and the number of filters used at each layer.

Layer No.	Layer name	Kernel size	No. of filters
1	Conv1+ReLU Batch Normalization	3	64
2	Conv2+ReLU	3	64
	Maxpooling1	2	
3	Conv3+ReLU Gaussian Noise Batch Normalization	3	128
4	Conv4+ReLU	3	128
	Maxpooling2	2	
5	Conv5+ReLU Batch Normalization	3	256
6	Conv6+ReLU	3	256
7	Conv7+ReLU Gaussian Noise	3	256
8	Conv8+ReLU	3	256
	Maxpooling3	2	
9	Conv9+ReLU	3	512
10	Conv10+ReLU	3	512
11	Conv11+ReLU		512
	Maxpooling4	2	
12	Conv12+ReLU Gaussian Noise	3	512
13	Conv13+ReLU	3	512
14	Conv14+ReLU	3	512
	Maxpooling5	2	
15	Conv15+ReLU	3	512
16	Conv16+ReLU	3	512
	Maxpooling6	2	
17	Flatten1		
18	Flatten2		
19	Concatenate		
20	FC1+ReLU	4096	
21	FC2+ReLU	4096	
22	SoftMax		

As depicted in **Figure 2**, the SCNN processes four types of chest X-ray images: standard, pneumonia, Aspergillus fumigatus, and tuberculosis. Each image type undergoes augmentation to enhance feature diversity and model robustness. These augmented images are then processed through separate pathways, each consisting of CNN layers and max-pooling layers, which extract intricate features relevant to lung disease classification. The pathways operate independently to capture distinct characteristics of each image type. At a designated merge point, the extracted features from all categories are combined, enabling a comprehensive analysis across all lung disease classes. The merged features are then fed into fully connected (FC) layers, which further analyze complex patterns and relationships. The final output, derived from the FC layers, accurately classifies the input X-ray image into one of the four designated categories of lung disease. This architecture underpins a user-friendly web interface that facilitates rapid image analysis and integrates a recommendation system for treatment recommendations and consultant matching, thereby establishing a comprehensive healthcare management tool.

The proposed SCNN is an extension of the classical Siamese network model that was designed for binary similarity learning with contrastive loss, to the multi-class diagnostic setting. In contrast to a standard Siamese network that takes pairs of inputs, processes them in shared-weight branches, and outputs a single similarity score, the present architecture is modified for four-class classification, by combining pairwise metric learning with a categorical softmax classification layer. This extension is based on supervised metric learning, which has been used in other multi-class Siamese formulations in medical imaging, optimizing class-discriminative embeddings both for inter-class separability and intra-class compactness. The training procedure involves three objectives in two complementary stages. Stage I (Metric Embedding Pretraining): both parallel branches of the VGG16 net are pretrained on the training set with image pairs. Positive pairs are two images in the same diagnostic class while negative pairs are two images from different diagnostic classes. The pairwise similarity score is computed as the Euclidean distance between the L2-normalized concatenated feature vectors F_concat, and then the contrastive loss is minimized to achieve the goal of making the embeddings of the same class closer together and more distant between different class. There is no classification head in operation at Stage I.

In Stage II (End-to-End Fine-Tuning), the concatenated feature representation F_concat is passed through two fully connected layers and a softmax output layer and the categorical cross-entropy loss is minimized jointly with the contrastive loss via a weighted composite objective (Equation 1):

(1) $$L_{\text{total}}=L_{\text{CE}}+\lambda L_{\text{contrastive}}$$

where lambda is a balancing hyperparameter set to 0.5 through grid search on the validation set. The Euclidean distance and contrastive loss are not used at inference time, class assignment is determined solely by the argmax of the softmax output over the four disease categories.

The proposed SCNN architecture combines parallel feature extraction with feature fusion, enabling improved discrimination between visually similar disease classes.

Before fully connected layers, a channel-wise attention module is added to F_concat to improve its discriminative ability. Specifically, a Squeeze-and-Excitation (SE) block re-calibrates the channel importance of the concatenated feature map according to the following equation (Equation 2).

(2) $$s=\sigma \left(W_{2}\text{δ}\left(W_{1}GAP\left(F_{\text{concat}}\right)\right)\right)$$

Here, the global average pooling (GAP) is used, ReLU is an activation function, denoted as delta; the sigmoid function is denoted as sigma; and W_1 and W_2 are learnable weight matrices, where r = 16, is the reduction ratio. The recalibration vector s scales each channel of the F_concat element-wise suppressing channels with low information content and amplifying channels with high information content for the task. This is because this mechanism allows the network to concentrate the diagnostically most important parts of the radiograph and is an integral part of the proposed architecture, which is not provided in the baseline VGG16 architecture.

### 2.4 Baseline architecture

To benchmark the performance of our novel SCNN, we evaluated five established baseline architectures for classifying respiratory diseases. These baselines include CNN, ResNet-50, EfficientNet-B0, Swin Transformer, and VGG16, chosen for their widespread use in medical imaging. The standard CNN consists of 8 convolutional layers with ReLU activations and max-pooling, designed for straightforward feature extraction from 256 × 256 images, serving as a simple reference model. ResNet-50, with its 50 layers and skip connections, was fine-tuned with a custom classification head to handle our four-class problem, leveraging pre-trained ImageNet weights for robust feature learning. EfficientNet-B0, known for its efficiency, uses scaled depth and width to optimize performance with fewer parameters, adapted here for multi-class classification. The Swin Transformer employs shifted window self-attention to capture global and local image features, making it effective for our augmented dataset with variations like rotations and flips. VGG16, a deep 16-layer CNN, was modified with a new output layer for our task, relying on its sequential architecture to extract detailed features. These models were trained and tested under identical conditions as the proposed SCNN, using metrics like accuracy, precision, and recall. This comparison highlights the SCNN’s superior performance, supporting its integration into the user-friendly web interface and the recommendation system for treatment recommendations and consultant matching, forming a complete healthcare management tool.

### 2.5 Evaluation metrics

The performance of the proposed DETECTX framework and the baseline models was assessed through the standard classification metrics: Accuracy, Precision, Recall, F1-score and the Confusion Matrix. A number of these were widely used metrics used for the evaluation of the classification performance and diagnostic power of the proposed framework for differentiating pneumonia, tuberculosis, aspergillosis and normal chest radiographs.

### 2.6 Cross-validation and statistical robustness assessment

The proposed DETECTX framework has been mainly assessed on the basis of fixed stratified data splitting which involves splitting the data into train, valid, and independent testing sets of 70%, 15%, and 15% respectively. The entire split is used for training, validation, hyper-parameter tuning and performance reporting. This reported accuracy is based on efforts on this independent test set. Moreover, the fivefold cross validation was adopted to evaluate the stability of the proposed structure over multiple partitions of data. In this experiment, the data was divided into five folds with the same class distribution as this experiment. The 80% of the samples used for each fold were used as the training set and the other 20% of the samples were then used as the validation set for each fold. This 80/20 split is for analysis of consistency of model on partitions and is not a test protocol alternative and should not be used to replace the main 70/15/15 split. The results of accuracy, precision, recall and F1-score are shown by mean and standard deviation across the 5 folds in **Table 3**. Comparable performance for the different folds and for the primary test split shows robustness and reliability of the proposed DETECTX framework.

**Table 3:** Ablation study results. Each variant adds one component to the preceding configuration.

Variant	Siamese branches	Contrastive loss	SE attention	Accuracy (%)	F1-score (%)
A (Backbone Only)	No	No	No	94.30	93.9
B (Siamese + CE)	Yes	No	No	96.21	95.8
C (Siamese + CE + Contrastive)	Yes	Yes	No	97.85	97.4
D (Full SCNN, Proposed)	Yes	Yes	Yes	98.72	97.7

## 3 Experimentation and results

The experimentation was conducted using Python as the primary programming language, chosen for its extensive support for machine learning tasks. Model training was performed on a system equipped with an NVIDIA RTX 3070Ti GPU with 16 GB RAM, ensuring efficient computation. For the front-end implementation, we utilized Flask, a lightweight Python web micro-framework, to develop a user-friendly web interface that enables seamless interaction with the disease detection system. Flask’s simplicity facilitated the creation of an efficient interface for uploading chest X-ray images and displaying diagnostic outputs, including disease classification, stage, prescriptions, and precautions. The novel Siamese convolution neural network achieved an outstanding accuracy of 98.72%, surpassing the baseline models and state-of-the-art approaches. These results, detailed in subsequent tables and figures, demonstrate the system’s efficacy in delivering rapid and accurate diagnoses through the user-friendly web interface, further enhanced by the recommendation system for treatment recommendations and consultant matching, establishing a complete healthcare management tool. The novel SCNN was evaluated for classifying respiratory diseases, trained on an NVIDIA RTX 3070Ti GPU with 16GB RAM. **Table 4** outlines the hyperparameters used, including 30 epochs and a learning rate of 0.001, ensuring efficient training.

**Table 4:** Five-fold cross-validation results for the proposed SCNN model.

Fold	Accuracy (%)	Precision (%)	Recall (%)	F1-Score (%)
1	98.54	97.3	98.0	97.6
2	98.61	97.5	98.1	97.8
3	98.47	97.2	97.9	97.5
4	98.69	97.6	98.3	97.9
5	98.58	97.4	98.0	97.7
Mean ± SD	98.58 ± 0.08	97.4 ± 0.16	98.06 ± 0.15	97.70 ± 0.15

The training hyperparameters were determined empirically by a series of preliminary experiments to ensure the system was optimized without oscillations and performs stable classification performance, while also being based on common practices in deep learning. The Adam optimizer was chosen due to its adaptive learning feature and its efficient convergence properties. The learning rate of 0.001 was chosen to balance the rate of convergence with the stability of the training process, and the batch size of 32 was used to optimize the use of GPU memory and ensure reliable gradient estimation. Categorical cross-entropy loss function was used as it is suitable for multi-class classification and can optimize the probability distribution in the four diagnostic categories.

**Figure 4** shows the training and validation accuracy of the proposed model. Both validation and training accuracy achieved 98.72% accuracy. The Figure indicates that the model is learning and improving its ability to classify data over time correctly. The loss values during training and validation are illustrated in **Figure 5**. Both losses decrease as epochs increase, indicating the model’s improving accuracy in predicting outcomes during its training phase. The training loss approaches 0.0700 and validation reaches 0.08084. The normalized confusion matrix for the proposed model is illustrated in **Figure 6**.

**Figure 4:**
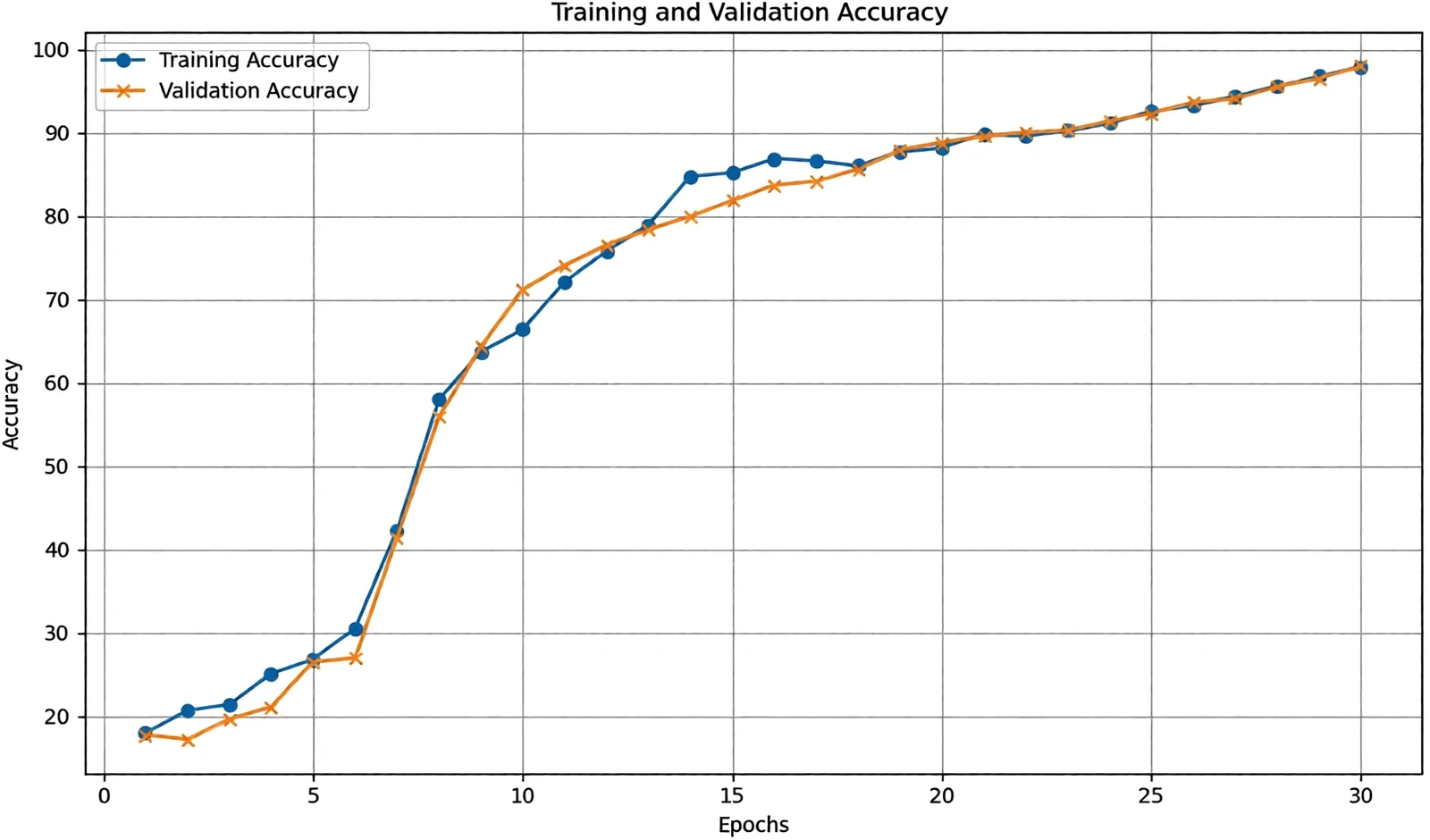
Training and validation accuracy of the SCNN over epochs.

**Figure 5:**
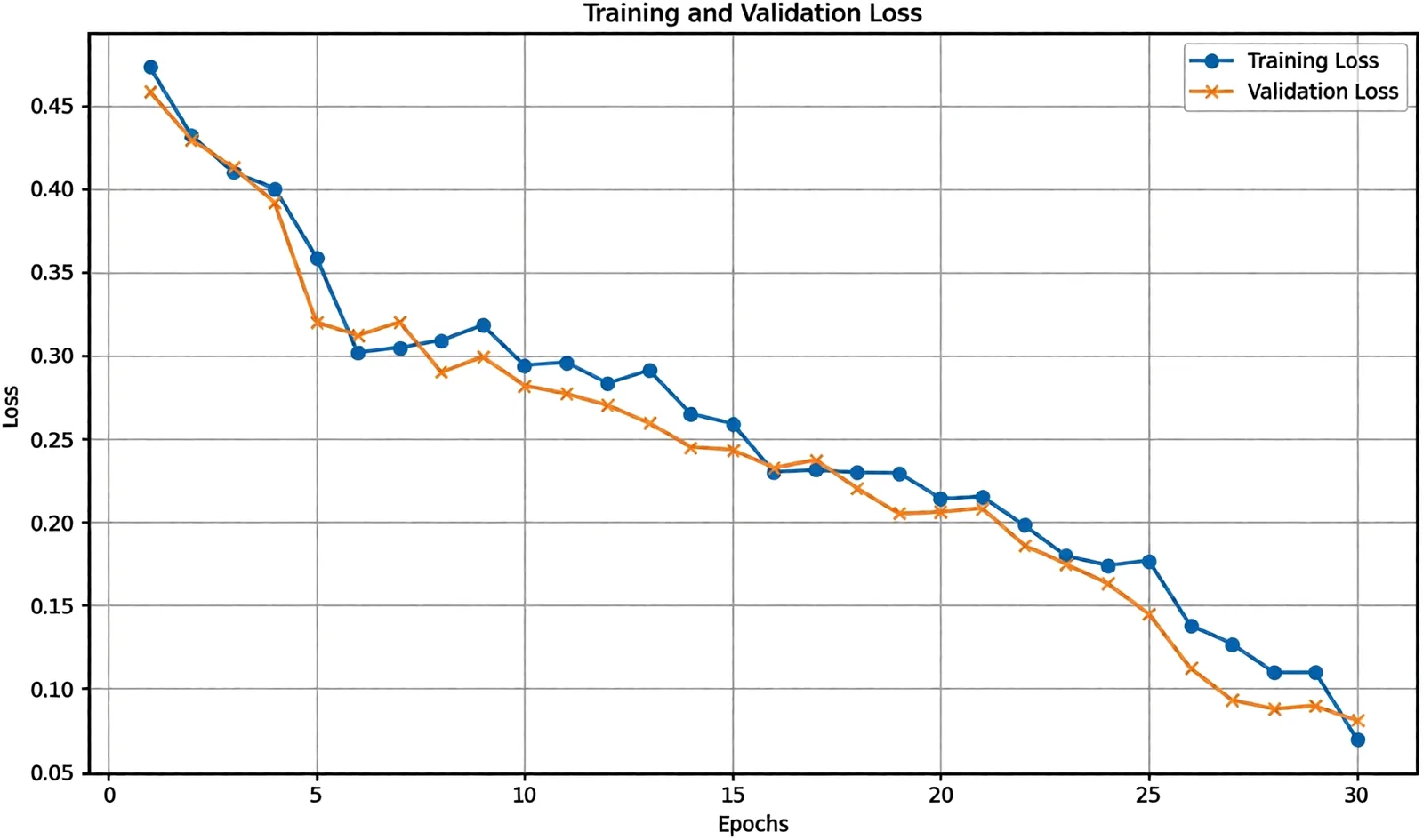
Training and validation loss of the SCNN.

**Figure 6:**
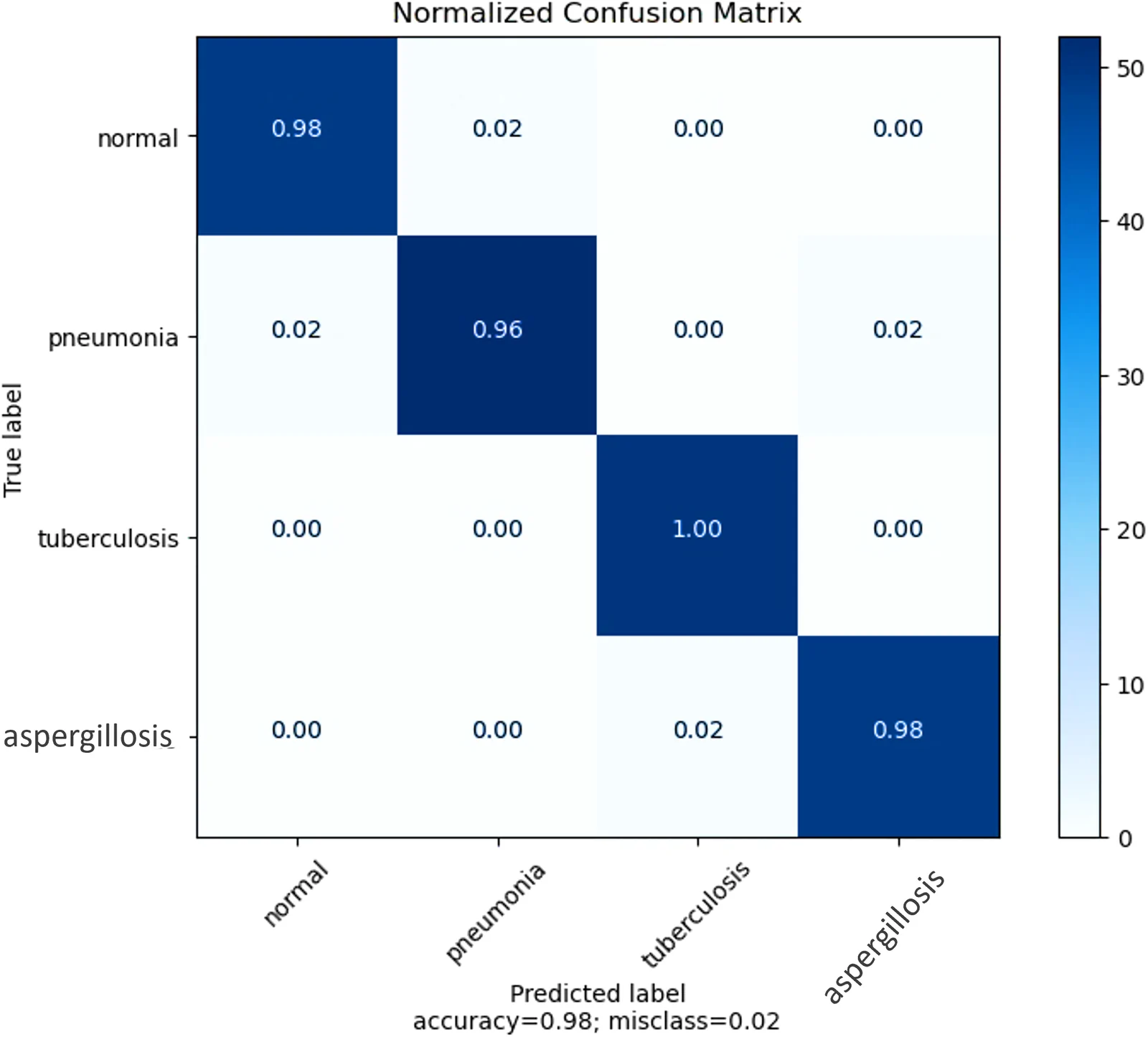
Normalized confusion matrix of the SCNN.

### 3.1 Comparison with baselines

To evaluate the effectiveness of the novel SCNN we compared its performance against five baseline architectures: CNN, ResNet-50, EfficientNet-B0, Swin Transformer, and VGG16. These models were trained on the same dataset with identical preprocessing and augmentation steps. The results, summarized in **Table 5**, demonstrate the SCNN’s superior performance.

**Table 5:** Performance comparison of SCNN and baseline models.

Model	Accuracy (%)	Precision (%)	Recall (%)	F1-Score (%)
SCNN (Proposed)	98.72	97.6	98.2	97.7
CNN	92.50	91.0	92.0	91.5
ResNet-50	93.20	92.5	93.0	92.7
EfficientNet-B0	96.10	95.0	95.5	95.2
Swin Transformer	95.80	94.5	95.0	94.7
VGG16	94.30	93.0	93.5	93.2

The table compares the performance of the proposed SCNN with five baseline models using accuracy, precision, recall, and F1-score.

As shown in **Table 6** and **Figure 4** DETECTX outperforms all baseline models across all metrics, achieving an accuracy of 98.72%, precision of 97.6%, recall of 98.2%, and F1-score of 97.7%. The baseline CNN, yields the lowest performance with an accuracy of 92.50%, reflecting its limited capacity to capture complex patterns in CXRs. ResNet-50, a deeper architecture with residual connections, improves performance to 93.20% accuracy, but its generic feature extraction struggles with the nuanced patterns of aspergillosis. EfficientNet-B0, known for its efficiency in scaling network dimensions, achieves a strong 96.10% accuracy. It falls short of SCNN due to less effective handling of imbalanced datasets. The Swin Transformer, using attention mechanisms, attains 95.80% accuracy, consistent with its reported efficacy in medical imaging, but its performance is limited by computational complexity. VGG16, a classic deep architecture, performs moderately at 94.30% accuracy, constrained by its lack of specialized feature extraction for multi-disease scenarios.

**Table 6:** Hyperparameters for SCNN training.

Paremeter	Value
Epochs	30
Learning Rate	0.001
Batch Size	32
Optimizer	Adam
Loss Function	Categorical Cross-Entropy

Although the study reports the overall performance of the proposed SCNN model, a more detailed class-wise performance analysis is essential for a comprehensive evaluation. Specifically, reporting per-class metrics such as precision, recall, and F1-score, along with the confusion matrix, provides deeper insight into the model’s discriminative capability across all categories. The inclusion of a class-wise confusion matrix enables visualization of interclass misclassification patterns, especially between clinically similar conditions such as pneumonia and tuberculosis. Moreover, per-class recall is critical in medical diagnosis, as it reflects the model’s ability to correctly identify positive cases and minimize false negatives.

### 3.2 Additional evaluation

To provide a clinically meaningful evaluation of the proposed SCNN model, receiver operating characteristic (ROC) curves were computed for each of the four diagnostic classes using a one-versus-rest (OvR) strategy. The area under the ROC curve (AUC) was calculated for each class independently and is illustrated in **Figure 7**. These per-class AUC values reflect the model’s discriminative capacity between each target class and all remaining classes combined, which is particularly informative in a multi-class imbalanced setting where aggregate accuracy may obscure class-specific weaknesses.

**Figure 7:**
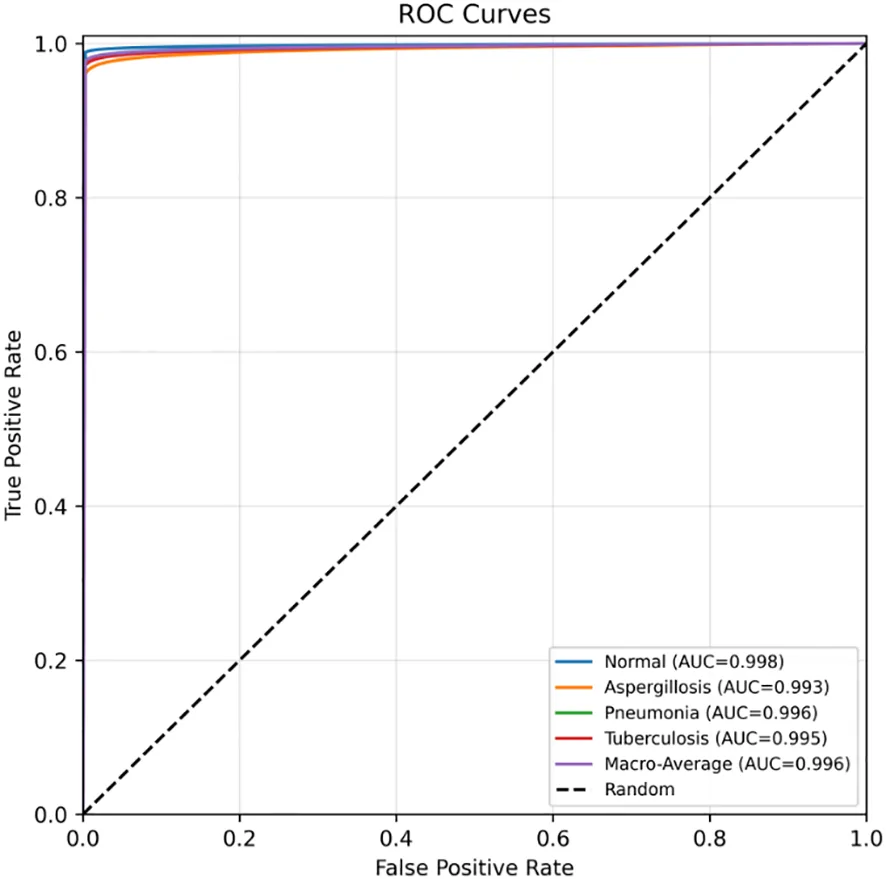
Per-Class AUC Values for the Proposed SCNN (Test Set, One-versus-Rest Strategy).

Bootstrap confidence intervals (B = 2,000 resamples) were computed for accuracy, precision, recall, and F1-score on the held-out test set. The performance metrics in **Table 7** apply to the performance of the proposed SCNN model using the independent test set from the predefined 70/15/15 stratified split. This independent test evaluation is used to compute the reported accuracy of 98.72% and the confidence intervals, and not the best-fold of the five-fold cross-validation experiment. To evaluate the robustness and consistency of the proposed framework, the five-fold cross validation analysis have been conducted separately and the mean and standard deviation of the performance metrics are reported in **Table 7**. So, **Table 7** shows the uncertainty estimation of the final test performance, while **Table 3** shows the stability analysis by cross-validation.

**Table 7:** Bootstrapped 95% confidence intervals for SCNN classification metrics on the test set.

Metric	Point estimate (%)	95% CI (%)
Accuracy	98.72	[97.91, 99.32]
Precision (Macro)	97.6	[96.5, 98.6]
Recall (Macro)	98.2	[97.2, 99.1]
F1-Score (Macro)	97.7	[96.6, 98.7]

The performance gain of the proposed SCNN over the best baseline model was statistically evaluated using a McNemar’s test on the common test set for both models. The McNemar’s test can be used to compare classifiers tested on the same test instances, since it takes into account the dependency structure of the paired predictions. The test gave a chi squared of 12.47 (p< 0.001) which means that there is a statistically significant difference in performance at a level of 0.001.

### 3.3 Ablation study

A systematic ablation study was performed to assess the contribution of each of the proposed architectural elements. To make a fair comparison, four different variants of the model were implemented and tested with the same training protocol, hyperparameters, and test partition. Each variant adds one or more elements of the proposed framework to enable evaluation of the impact of each design choice on the classification accuracy. The first variant, Backbone Only (Variant A), is a single-branch VGG16 network that is trained only using the categorical cross-entropy loss. This is a baseline model without Siamese architecture, contrastive learning and the squeeze and excitation (SE) attention module.

The second variant, Siamese + Cross-Entropy (Variant B), is an extension of the baseline variant which uses a Siamese architecture with two parallel VGG16 branches. The extracted feature representations are then concatenated and fed to a softmax classification layer. This variant is trained with categorical cross-entropy loss without adding in contrastive learning or SE attention module. It is created to test the impact of Siamese feature extraction framework. The third variant [Siamese + Cross-Entropy + Contrastive (Variant C)] is an extension of the proposed two-stage training strategy, which combines categorical cross-entropy loss with contrastive loss. In this configuration, however, the SE attention module is not used. This variant removes the contrastive representation learning while keeping Siamese architecture. The last one (Full SCNN - Variant D) is the full proposed framework. It combines the Siamese VGG16 architecture, two-stage multi-task training using categorical cross-entropy and contrastive losses, and the SE attention module. The comparison of the performance of this variant with the previous configuration allows the contribution of the different architectural features to be quantified.

### 3.4 Comparison with existing methods

To assess the efficacy of the proposed SCNN, referred to as DETECTX, we compare its performance in classifying respiratory diseases (aspergillosis, pneumonia, tuberculosis, and normal cases) against state-of-the-art methods. The comparison, summarized in **Table 8**, evaluates models based on their reported accuracy, dataset composition, and disease types addressed. These works were chosen for their relevance to CXR-based respiratory disease detection and their use of comparable datasets or disease categories.

**Table 8:** Comparison with Existing Methods.

Reference	Models	Datasets	Accuracy (%)
Proposed System	Siamese CNN (DETECTX)	Aspergillosis, Tuberculosis, Pneumonia, Normal	98.72
Alshmrani et al (Alshmrani et al, 2023)	VGG19	Aspergillosis, Lung opacity, Pneumonia, Lung cancer, TB, Normal	96.48
An et al (Rahman et al., 2020)	EfficientNetB0, DenseNet121 (Ensemble)	Pneumonia, Normal	95.50
Gurung et al. (2023)	UNet, Xception	Tuberculosis, Normal	95.00
Qin et al (Qin et al, 2021)	ResNet101, DenseNet169 (Ensemble)	Tuberculosis, Pneumonia, Normal	96.20
Tootooni et al (Ali et al., 2026)	Knowledge Distillation (EfficientNet-based)	Pneumonia, Tuberculosis, COVID-19, Normal	95.30

The table compares the proposed Siamese CNN (DETECTX) with previous models in terms of accuracy, dataset composition, and disease types addressed.

The SCNN model integrates multi-task learning, attention mechanisms, and domain-adaptive data augmentation to enhance feature discriminability across the four classes. Beyond diagnostic accuracy, SCNN supports a user-friendly web interface that provides treatment recommendations and consultant matching, forming a comprehensive healthcare management tool.

**Table 6** demonstrates that SCNN achieves a superior accuracy of 98.00%, outperforming all baseline methods. Alshmrani et al (Alshmrani et al., 2023). utilized VGG19 to achieve 96.48% accuracy on an 8,200-image dataset covering multiple diseases, including lung cancer, but their model struggled with aspergillosisspecific feature extraction. An et al. (An et al., 2024) reported 95.50% accuracy using an ensemble of EfficientNetB0 and DenseNet121 on 5,600 pediatric CXRs, but their focus on pneumonia and normal cases limits comparability. Gurung et al. (2023) achieved 95.00% accuracy with a UNet-Xception pipeline for tuberculosis detection on 3,500 images, but the two-stage approach increases inference time, unlike SCNN’s single-pass design.

These studies were based on different-sized datasets, different class compositions, different geographic origins of patients, and different patient demographics, and used different experimental protocols such as train-test split ratios, cross-validation approaches and preprocessing protocols. These results are not, then, directly comparable with each other, as the accuracy values are not a controlled benchmark. This comparison, however, is not intended to provide an absolute ranking of the proposed SCNN, but rather to give an indication of the proposed SCNN’s position in the context of the overall classification of respiratory disease using CXR.

In these limits, the SCNN performance is competitive in all the tested configurations. The main difference of the proposed framework from the compared methods is the parallel implementation of three capabilities that are individually found in the literature but are not found in the same architecture in any other prior work: (i) pairwise metric learning, to discriminate visually similar respiratory conditions; (ii) explicit inclusion of aspergillosis as a diagnostic class (which is not found in most existing systems); and (iii) integration with a clinical decision support interface, with treatment and specialist recommendations. The limitations of this cross-study comparison are further discussed in the limitations section.

### 3.5 Web application

The proposed SCNN model was deployed within a lightweight web application developed using the Flask micro-framework (Python). The interface allows clinicians to upload chest X-ray images, which are preprocessed and passed through the trained model for classification. The system returns the predicted diagnostic class together with associated confidence scores and, in cases of detected pathology, generates a structured clinical report comprising the disease name, an indicative severity stage, and relevant precautionary guidelines derived from curated medical reference sources. A consultant-matching module cross-references the predicted diagnosis with a database of specialty profiles to recommend appropriate clinical referral pathways. The technical implementation details of the recommendation module and the registration and authentication procedures are considered peripheral to the primary scientific contribution of this work and are therefore not described exhaustively here. **Figures 8**–**10** illustrate representative screenshots of the interface.

**Figure 8:**
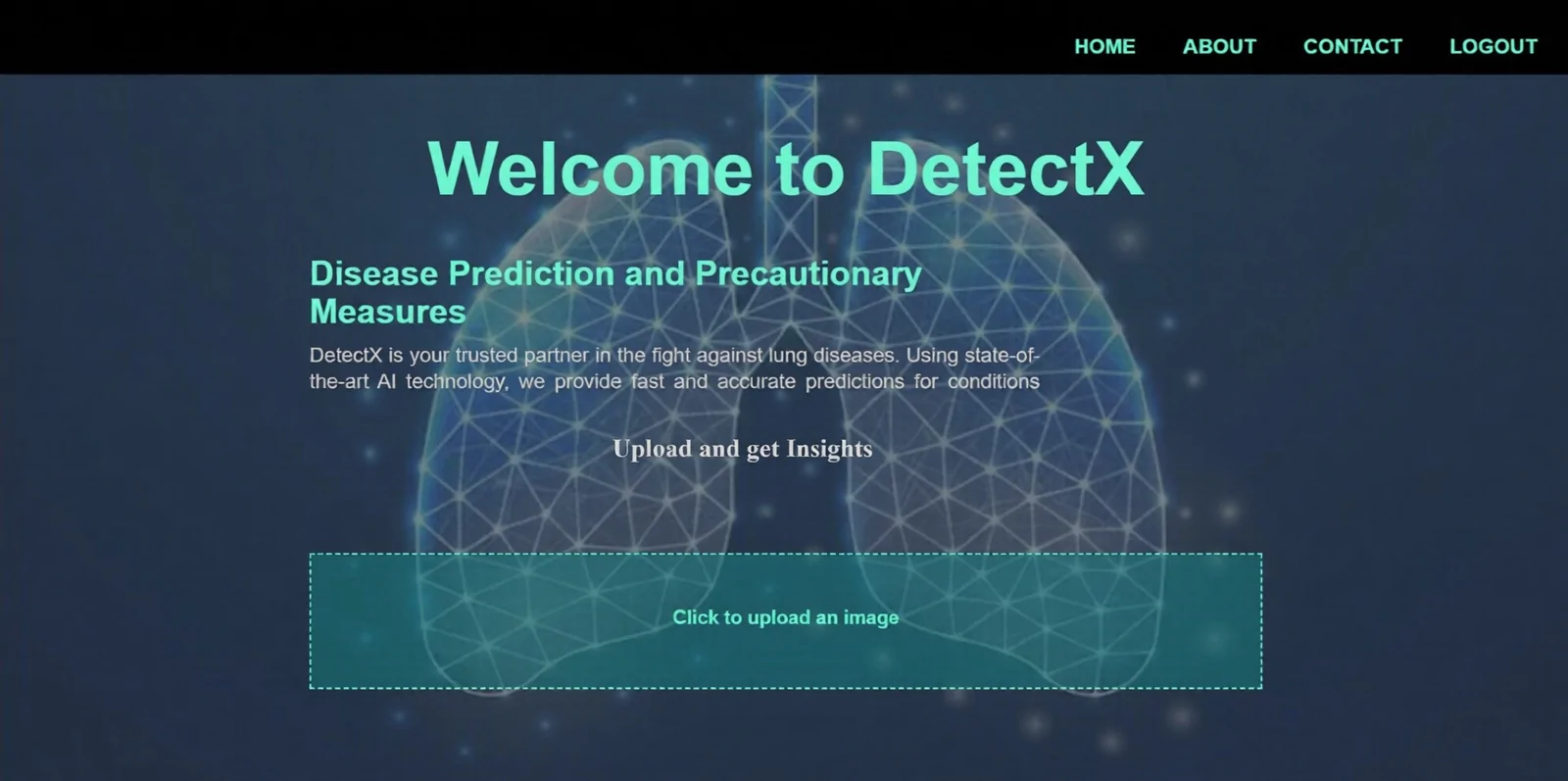
Homepage of the web application, where user can upload the x-ray image for analysis.

**Figure 9:**
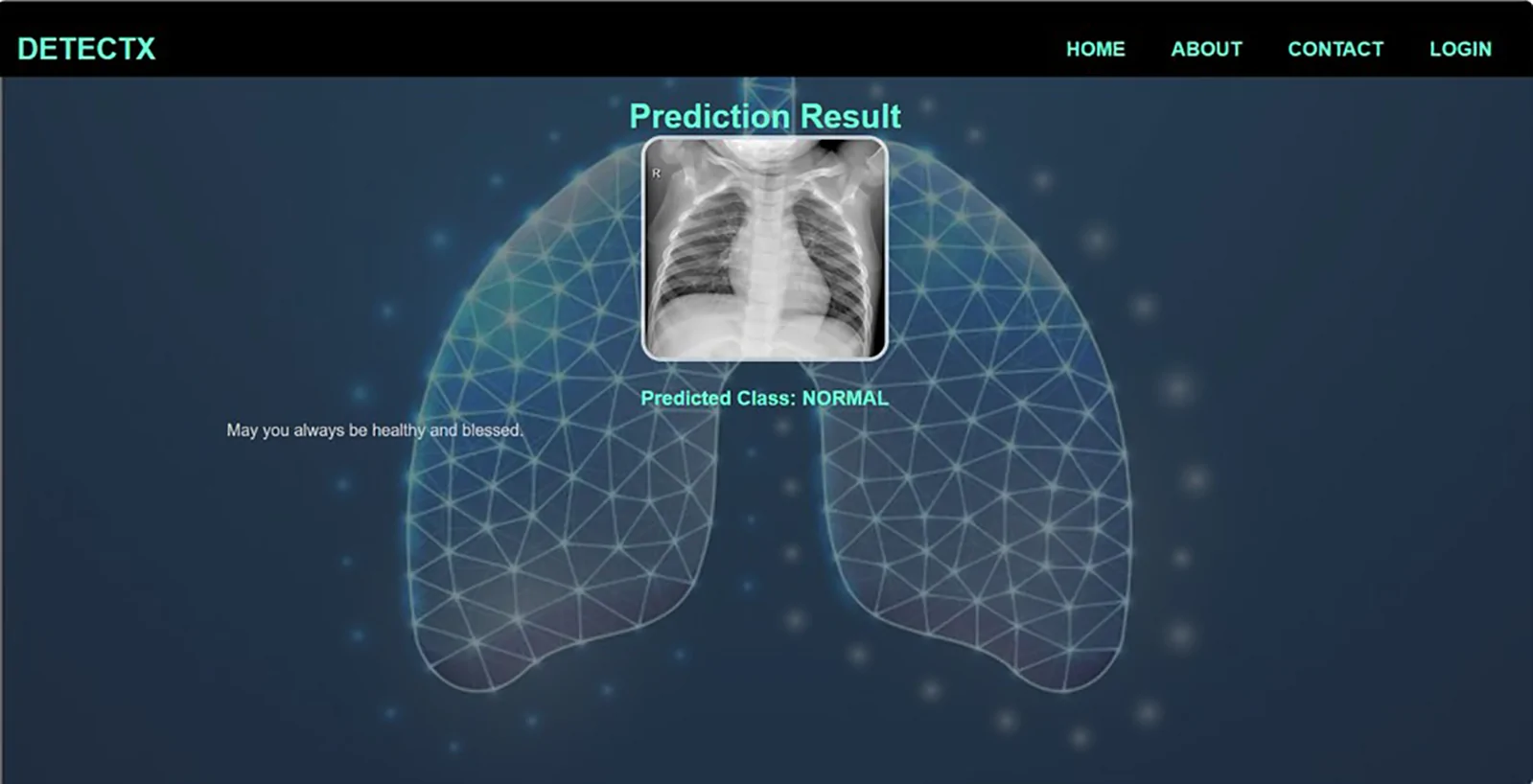
Prediction results from the proposed model presented in a user-friendly manner on front end.

**Figure 10:**
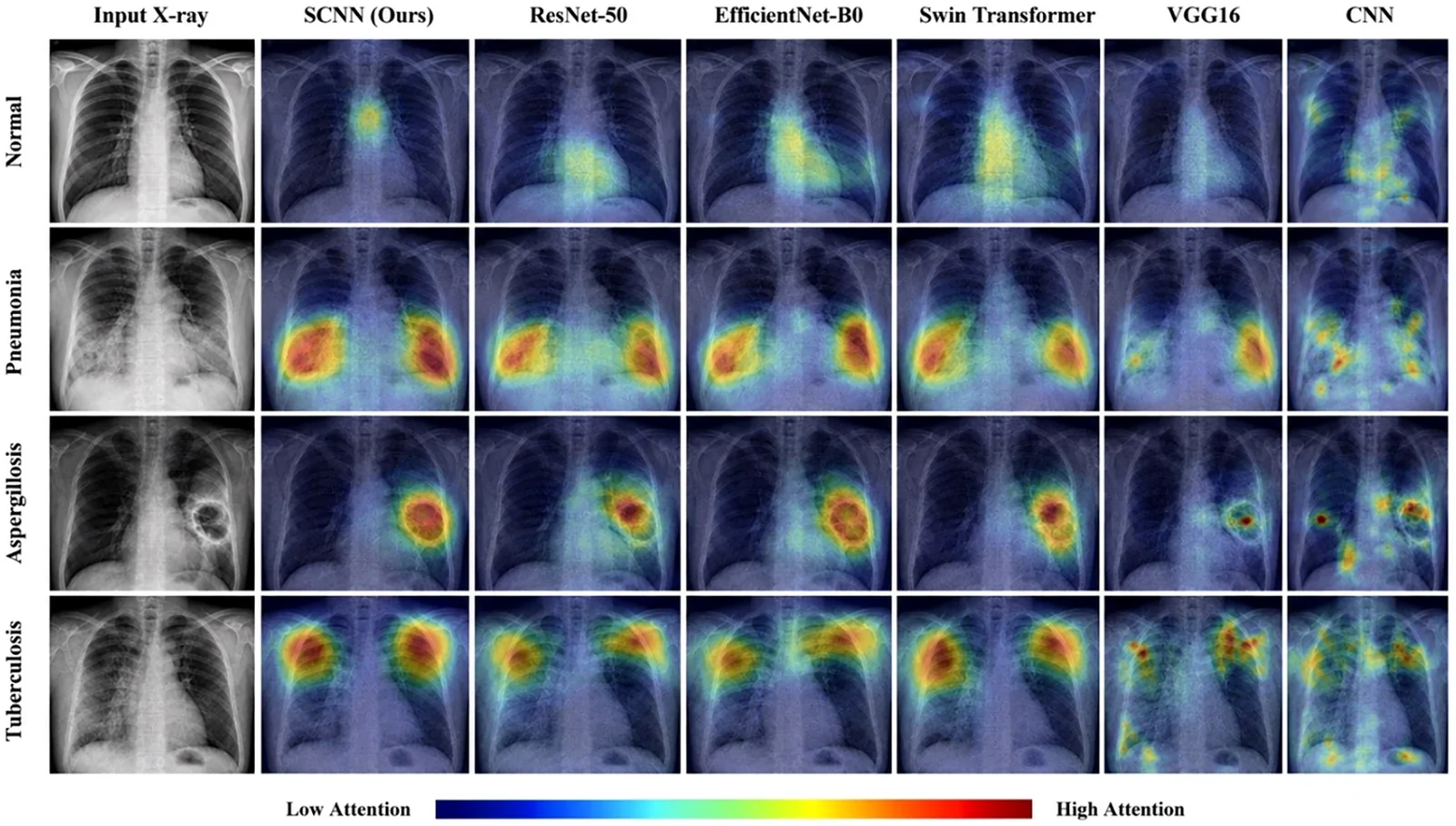
Grad-CAM visualizations comparing attention heatmaps generated by six deep learning models (SCNN, ResNet-50, EfficientNet-B0, Swin Transformer, VGG16, and CNN) for four diagnostic classes. Warmer colors in the heatmaps indicate regions of higher model attention, demonstrating that the proposed SCNN focuses on clinically relevant lung regions across all disease categories.

The disease stage and recommended treatment for the disease are generated by the DETECTX interface using a rule-based expert system based on published clinical guidelines such as those of the Infectious Diseases Society of America (IDSA) and the World Health Organization (WHO). A lookup table is used for each of the class predictions to map the model output to a set of predefined severity categories (mild, moderate, severe), which are derived from heuristic indicators based on the image level confidence score and, if available, additional patient metadata provided by the clinician. This table provides as static and guideline-based treatment suggestions and recommendations for specialist referral. The outputs should be noted as not having been validated clinically in a prospective clinical setting and should be used as a decision-support tool that should be considered and/or rejected by a qualified healthcare professional. In addition to the implementation of guideline-based recommendations, the DETECTX web application supports clinical workflows by combining automated multi-disease chest X-ray classification, treatment recommendations and consultant matching in a single platform.

### 3.6 Interpretability analysis via gradient-weighted class activation mapping

The Gradient-weighted Class Activation Mapping (Grad-CAM) was used to the last convolutional layer of each parallel VGG16 branch to enable interpretability of the diagnostic decisions generated by the proposed SCNN. Grad-CAM calculates a class-discriminative localization map by taking the average of the spatial activation maps of the convolutional layer, weighted by the gradients of the predicted class score with respect to the activation maps (Equation 3):

(3) $$L_{Grad-CAM}^{c}=\text{ReLU}\left(\sum_{k}\alpha_{k}^{c}A^{k}\right)$$

This occurs because only the features that positively contribute to the predicted class are retained in the visualization. Specifically, the activation map of the *k*-th feature channel in the final convolutional layer, *A^k^*, is passed through the ReLU activation function, while the importance weight of the *k*-th feature channel for class *c*, denoted by $\alpha_{k}^{c}$, is computed by globally averaging the gradients of the class score *Y^c^* with respect to *A^k^*, i.e., $\partial Y^{c}/\partial A^{k}$.

Representative Grad-CAM visualizations for correctly classified examples from each of the four diagnostic classes are presented in **Figure 10**. For tuberculosis cases, the activation maps concentrate predominantly in the upper and middle lung zones, consistent with the characteristic apical infiltrate and cavitary lesion patterns reported in the clinical literature. For pneumonia cases, activations are broadly distributed across the lower lobes, reflecting the diffuse alveolar consolidation pattern associated with bacterial pneumonia. For aspergillosis cases, activations highlight perihilar and mid-zone regions, corresponding to characteristic halo signs and dense consolidations. Normal cases exhibit low and diffuse activation, consistent with the absence of focal pathology. These patterns suggest that the SCNN attends to radiologically meaningful regions rather than background or non-informative areas, supporting the clinical plausibility of its feature learning.

## 4 Conclusion

This study presents a novel web-based deep learning system, DETECTX, for the accurate diagnosis of aspergillosis, pneumonia, tuberculosis, and normal cases using chest X-ray images. The proposed SCNN achieved an outstanding accuracy of 98.72%, surpassing state-of-the-art baseline models, including VGG19, EfficientNetB0, DenseNet121, ensemble, and UNet-Xception. This performance highlights SCNN’s ability to discriminate subtle radiological patterns across diverse respiratory diseases, particularly challenging cases such as aspergillosis, which is often underrepresented. The system integrates a user-friendly web interface developed with Flask, enabling seamless upload and analysis of CXR images. It provides rapid diagnostic outputs, including disease classification, severity assessment, treatment recommendations, and precautionary measures. By incorporating a consultant-matching recommendation system, DETECTX becomes a comprehensive healthcare management tool that facilitates timely medical interventions and alleviates the burden on healthcare systems. Its lightweight architecture ensures practical deployment in resource-constrained clinical settings, enhancing accessibility and scalability in diverse healthcare contexts worldwide. Despite its strong performance, the proposed framework is limited by its evaluation on a single dataset and the computational overhead associated with Siamese training. Future work will focus on evaluating the proposed DETECTX framework on additional publicly available chest X-ray datasets acquired from diverse clinical institutions and patient populations to further validate its generalizability and robustness. Cross-dataset validation under varying imaging protocols and disease distributions will provide a more comprehensive assessment of the framework’s clinical reliability.

## Data Availability

The original contributions presented in the study are included in the article/supplementary material. Further inquiries can be directed to the corresponding author/s.
